# Prevalence and genotypic identification of *Cryptosporidium* spp. and *Enterocytozoon bieneusi* in captive Asiatic black bears (*Ursus thibetanus*) in Heilongjiang and Fujian provinces of China

**DOI:** 10.1186/s12917-020-02292-9

**Published:** 2020-03-10

**Authors:** Sheng-Nan Wang, Yun Sun, Huan-Huan Zhou, Gang Lu, Meng Qi, Wei-Shi Liu, Wei Zhao

**Affiliations:** 1 College of Wildlife Resources, Northeast Forestry University, Harbin, 150040 China; 2 The Animal Husbandry and Veterinary Research Institute of HaiBei Tibetan Autonomous Prefecture, Xining, 810299 HaiBei Tibetan Autonomous Prefecture China; 3 Department of Pathogenic Biology, Hainan Medical University, Haikou, Hainan 571199 China; 4 Key Laboratory of Tropical Translational Medicine of Ministry of Education, Hainan Medical University, Haikou, Hainan 571199 China; 5 Hainan Medical University-The University of Hong Kong Joint Laboratory of Tropical Infectious Diseases, Hainan Medical University, Haikou, Hainan 571199 China; 6 College of Animal Science, Tarim University, Alar, Xinjiang 843300 China; 7 Department of Parasitology, Wenzhou Medical University, Wenzhou, 325035 China

**Keywords:** *Cryptosporidium*, *Enterocytozoon bieneusi*, Bear, Genotypic, Identification

## Abstract

**Background:**

*Cryptosporidium* and *Enterocytozoon bieneusi* are two important pathogens with zoonotic potential that cause enteric infections in a wide range of hosts, including humans. Both are transmitted from animals to humans by direct contact or through contaminated equipment. Bears are frequently found in Chinese zoos as ornamental animals as well as farmed as commercial animals, and are therefore in close contact with zoo- or farm-keepers, but the prevalence and zoonotic potential of *Cryptosporidium* and *E. bieneusi* in bears is poorly understood. In this study, we aimed to provide data on the occurrence and genetic diversity of *Cryptosporidium* and *E. bieneusi* in Asiatic black bears from Heilongjiang and Fujian, China. From May 2015 to December 2017, 218 fresh fecal specimens were collected from captive Asiatic black bears in Heilongjiang (*n* = 36) and Fujian (*n* = 182), China. *Cryptosporidium* and *E. bieneusi* were examined by PCR amplification of the partial small subunit of ribosomal DNA (SSU rDNA) and the internal transcribed spacer (ITS) region of rDNA, respectively. *C. andersoni-*positive isolates were subtyped through PCR analysis of the four minisatellite/microsatellite (MS1, MS2, MS3 and MS16) loci.

**Results:**

The overall prevalence of *Cryptosporidium* and *E. bieneusi* were 2.4% (4/218) and 6.4% (14/218), respectively, with 2.8% (1/36) and 22.2% (8/36) in the Heilongjiang Province, and 1.6% (3/182) and 3.3% (6/182) in the Fujian Province. Sequence analysis confirmed the presence of *Cryptosporidium* species: *C. andersoni* (*n* = 3) and a genotype termed *Cryptosporidium* rat genotype IV (*n* = 1). All three identified *C. andersoni* belonged to the MLST subtype A4, A4, A4, A1. Two known *E. bieneusi* genotypes D (*n* = 4) and SC02 (*n* = 10) were identified, both of which belong to zoonotic Group 1.

**Conclusions:**

This is the first report of *C. andersoni* and *Cryptosporidium* rat genotype IV in bears. The discovery of the zoonotic potential of *E. bieneusi* genotype D in bears highlights its significant zoonotic potential and potential threat to human health.

## Background

*Cryptosporidium* and *Enterocytozoon bieneusi* are two important pathogens with zoonotic potential that cause diarrhea in humans and a variety of animal species [1, 2]. For humans, both are responsible for self-limiting diarrhea in immune competent individuals, and severe life-threatening diarrhea in patients with immune deficiency [3, 4]. *Cryptosporidium* oocysts and *E. bieneusi* spores, the infective stage, are ubiquitous in the environment. Both humans and animals can be infected through direct contact with an infected host, or through indirect ingestion of oocyst-contaminated water or food [2, 3]. Current epidemiological data of *Cryptosporidium* and *E. bieneusi* have raised public health concerns about their zoonotic nature in addition to water- and food-borne transmission [2, 3]. Tracing the sources of contamination and elucidating the transmission routes of *Cryptosporidium* and *E. bieneusi* are important steps in adequately controlling human infections.

The use of PCR-based molecular tools for the characterization of *Cryptosporidium* and *E. bieneusi* have improved our understanding of their epidemiology, providing information on the host distribution of various species/genotypes and transmission routes/sources [4, 5]. To date, 39 species of *Cryptosporidium* and more than 70 genotypes having been described [5, 6]. Amongst them, 21 *Cryptosporidium* species/genotypes have been identified in humans, of which *C. hominis* and *C. parvum* are the most common, causing > 90% of human cryptosporidiosis cases [5]. Other species including *C. meleagridis*, *C. ubiquitum*, and *C. cuniculus* have emerged as zoonotic pathogens and have received more attention based on their higher number of sporadic cases and resultant human cryptosporidiosis outbreaks [5]. Contact with animals is a risk factor for disease [7].

Most current genotyping studies are based on the sequence analysis of 243 bp of the ITS region, regarded as the standard method for the detection and identification of *E. bieneusi* genotypes [8]. To date, more than 400 ITS genotypes have been reported that can unambiguously be placed into 11 distinct clades by phylogenetic analysis [9, 10]. A large group (Group 1) comprises genotypes from both humans and animals, showing its zoonotic nature; the other eight groups (Groups 2 to 11) mainly include genotypes from specific hosts or wastewater, showing host adaption [10, 11]. To improve our understanding of the epidemiology of human microsporidiosis and strengthen the knowledge of *E. bieneusi* populations and their human transmission, epidemiological surveys should focus on genotyping *E. bieneusi* isolates from animal hosts that are in close contact with humans.

Asiatic black bears are frequently found in Chinese zoos as commercial and ornamental animals, and are therefore in close contact with zoo-keepers [12]. As a result of the high-density feeding environment in zoos and potential exposure to bear feces, infective spores of *E*. *bieneusi* from Asiatic black bears pose a potential risk to other animals and public health [13]. Currently, only two reports described *E. bieneusi* infections in these animals in China and no information about prevalence and genotypes of *Cryptosporidium* infection in Asiatic black bears in mainland of China is available [12, 13]. The health of bears is thus important in terms of management objectives and public health. The aim of this study was to determine the prevalence of natural *Cryptosporidium* and *E. bieneusi* infections in bears from the Heilongjiang and Fujian Province, China, to genotype *Cryptosporidium* and *E. bieneusi* isolates by sequence analysis of PCR products of the SSU rRNA and the ITS region of the rRNA gene, respectively. We further assessed potential zoonotic transmission by phylogenetic analysis based on the neighboring-joining method.

## Results

### Occurrence of *Cryptosporidium* and *E. bieneusi*

A total of 4/218 (1.8, 95% CI: 0–3.6%) and 14/218 (6.4, 95% CI: 3.1–9.7%) fecal specimens were positive for *Cryptosporidium* and *E. bieneusi*, respectively, and the differences between the overall prevalence values of the two pathogens were significant (*P* = 0.03, χ^2^ = 5.80). The prevalences of *Cryptosporidium* in bears was 2.8% (1/36) in Heilongjiang and 1.6% (3/182) in Fujian, while the prevalence of *E. bieneusi* in bears was 6.4% (14/218) with 22.2% (8/36) in Heilongjiang and 3.3% (6/182) in Fujian (Table 1). There were significant differences in the infection rates of *E. bieneusi* amongst bears from Heilongjiang and Fujian (*P* = 0.006), but no differences in *Cryptosporidium* infection in the two farms was observed (*P* > 0.05).

**Table 1:** Prevalence and *Cryptosporidium* species/genotypes and *E. bieneusi* genotypes in bears according to age, gender and feeding mod

Group	Fujian					Heilongjiang				
	Examined No.	*E. bieneusi*		*Cryptosporidium*		Examined No.	*E. bieneusi*		*Cryptosporidium*	
		Positive No. (%)	Genotype (n)	Positive No. (%)	Species/genotype (n)		Positive No. (%)	Genotype (n)	Positive No. (%)	Species/genotype (n)
Age										
< 3(year)	17	1 (5.9)	D (1)	1 (5.9)	Rat genotype IV (1)	12	4 (33.3)	SC02 (4)	0	/
3–5(year)	122	5 (4.1)	D (3); SC02 (2)	0	/	–	–	–	0	/
> 5 (year)	43	0	/	2 (4.7)	*C. andersoni* (2)	24	4 (16.7)	SC02 (4)	1 (4.2)	*C. andersoni* (1)
Gender										
Male	118	4 (3.4)	D (2); SC02 (2)	3 (2.5)	Rat genotype IV (1); *C. andersoni* (2)	15	3 (20.0)	SC02 (3)	1 (6.7)	*C. andersoni* (1)
Female	64	2 (3.1)	D (2)	0	/	21	5 (23.8)	SC02 (5)	0	/
Feeding mode										
Alone	92	4 (4.3)	D (3); SC02 (1)	2 (2.2)	*C. andersoni* (2)	36	8 (22.2)	SC02 (8)	1 (2.8)	*C. andersoni* (1)
Group	90	2 (2.2)	D (1); SC02 (1)	1 (1.1)	Rat genotype IV (1)	–	–	–	0	/
Total	182	6 (3.3)	D (4); SC02 (2)	3 (1.6)	Rat genotype IV (1); *C. andersoni* (2)	36	8 (22.2)	SC02 (8)	1 (2.8)	*C. andersoni* (1)

### Genotyping and subtyping of *Cryptosporidium*

All four *Cryptosporidium-*positive specimens were successfully sequenced at the SSU rRNA locus. By sequence analysis, *C. andersoni* (*n* = 3) and *Cryptosporidium* rat genotype IV (*n* = 1) were identified. All three sequences of *C. andersoni* had 100% homology and were identical to DQ989573 from bactrian camels in Henan, China. The *Cryptosporidium* rat genotype IV sequence (MN726617) obtained was not reported previously and had 99.3% homology to the sequence (MG917670) of the isolate from brown rats in Heilongjiang, China.

All three *C. andersoni*-positive isolates were further subtyped through the amplification of four minisatellite/microsatellite loci (MS1, MS2, MS3 and MS16) and all were successfully amplified and sequenced. Only one MLST subtype (A4, A4, A4, A1) termed was identified. All four microsatellite/minisatellite sequences identified were described previously.

In this study, *C. andersoni* was only found in male animals aged ≥5 years, whilst *Cryptosporidium* rat genotype IV was identified in young male bears from Fujian (Table 1).

### Genotyping of *E. bieneusi*

By sequence analysis of the ITS region of 14 *E. bieneusi* isolates obtained in this study, two known genotypes were identified including D (*n* = 4) and SC02 (*n* = 10). The distributions of *E. bieneusi* genotypes in animals was dependent on age, gender, and feeding mode (Table 1). Genotype D was found solely in Fujian and SC02 were found both in Heilongjiang and Fujian. Genotypes were classified in Group 1, which represents a zoonotic group (data not show).

## Discussion

*Cryptosporidium* has only been reported in two captive Malayan sun bears (*Helarctos malayanus*) from Taiwan, China, in a black bear from Virginia, USA [14–16] and in 35 brown bears from the Slovak Republic [17]. The *Cryptosporidium* prevalence of 2.4% in this study was lower than the 55.6% reported in the Slovak Republic [17]. To date, only three studies of *E. bieneusi* in bears have been reported, two of which are from China [12, 13], with one study from the USA [18]. In China, the prevalence was 19.75% (80/405) and 27.4% (29/106) in Yunnan and Southwestern regions, respectively [12, 13], both of them were significantly lower than the prevalence of 40% (2/5) observed in wild black bears in New York City [11]. In fact, the bears in the studies from China are captive, while the animals in the study from USA are wild bears. Meanwhile, the number of the wild bears are limited (only five wild bears were examined). Here, the prevalence of *E. bieneusi* in bears was 6.4% (14/218) with 22.2% (8/36) in Heilongjiang and 3.3% (6/182) in Fujian. The observed differences in prevalence of *E. bieneusi* among different bears may be explained by variations in feeding density, geography, management systems, sample sizes, and climate, however, the possible influence of the mentioned factors above on the prevalence remains entirely unexplored, and further studies are still necessary to elucidate this aspect.

To date, only two studies have characterized *Cryptosporidium* in bears and only *C. parvum* and *Cryptosporidium* bear genotype have been identified in those animals [15, 16]. Xiao et al. (2000) identified the *Cryptosporidium* bear genotype in black bears and showed its close relationship to *C. canis* at the SSU rRNA and HSP-70 loci [15]. Duncan et al. (1999) detected *C. parvum* in tissue sections from the small intestine of black bear cubs found dead in Virginia (USA) [16]. In the present study, *C. andersion* and *Cryptosporidium* rat genotype IV were identified in bears, and *C. andersion* had the highest frequency and widest distribution. To the best of our knowledge, this was the first identification of *C. andersion* and *Cryptosporidium* rat genotype IV in bears, which provides useful information on the molecular epidemiology and control of *Cryptosporidium* infections in animals.

Although *C. andersoni* is widely considered a cattle-specific *Cryptosporidium* species, it’s occurrence in other animals and humans is increasing [18, 19]. To date, *C. andersoni* has been detected in cattle, yaks, sheep, goats, deer, rodents, houses, non-human primates, camels, and giant pandas [18, 20–29]. Contrasting with its occasional detection in other animals, *C. andersoni* is the common *Cryptosporidium* species in cattle, which appear its natural host [18]. In fact, *C. andersoni* is commonly observed in adult cattle and has been associated with gastritis, reduced milk yield, and poor weight gain. A limited number of studies have reported *C. andersoni* infections in humans from France, Malawi, Iran, England and Australia [19, 30–34]. However, recent studies reported that *C. andersoni* was found in 78 diarrheal patients from southern Assam, India [35], and 34 and 21 patients with diarrhea in Shanghai and Jiangsu of China [19, 36].

With the development of *Cryptosporidium* subtyping tools, more and more *C. andersoni* isolates were subtyped with a multilocus sequence typing (MLST) tool based on the MS1, MS2, MS3, and MS16 loci in China, those isolates were mainly from cattle as well as from yaks, sheep, horses, golden takin, camels and non-human primates [18, 24–29]. Sixteen MLST subtypes were identified. Amongst them A4, A4, A4, A1 was the predominant subtype in cattle from Henan, Jilin, Guangxi, Heilongjiang, Sichuan, and Shaanxi of China, and was found in non-human primates and horses from Qinling Mountains and Heilongjiang, China, respectively [18, 24, 27]. In this study, all three isolates of *C. andersoni* identified in bears had the A4, A4, A4, A1 subtype. The results showed that *C. andersoni* isolates in bears were likely to be transmitted from other animals, particularly cattle, but the real source of infection and transmission require further analysis.

*Cryptosporidium* rat genotype IV, previously named the *Cryptosporidium* W19 or *Cryptosporidium* W19 variant, have been recorded in rats including Asian house rats, brown rats, Edward’s long-tailed rats and muridae [37]. Meanwhile, epidemiology data suggests contamination of the water supplies evidenced by their detection in streams in the USA and raw water in the UK and China [38–40]. No occurrences of *Cryptosporidium* rat genotype IV in any animal species other than rats have been reported. This is the first report of *Cryptosporidium* rat genotype IV in bears, indicating that this genotype has a broader range of reservoir hosts than initially anticipated. The potential of *Cryptosporidium* rat genotype IV to cause disease in humans or livestock is unknown, and the source of *Cryptosporidium* rat genotype IV infection and its transmission dynamics now require further investigation. This will reveal the cross-species transmission potential of *Cryptosporidium* rat genotype IV in rats and other animals, including bears in China.

To date, 20 genotypes (D, SC01, SC02, CHB1, horse2, ABBA, ABB2 and MJ1 to MJ13) of *E. bieneusi* have been identified in bears, of which only genotype D has been shown to have human infective potential [11–13]. In this study, two known *E. bieneusi* genotypes, D and SC02, were identified. Genotype SC02 was identified in 71.4% of *E. bieneusi* isolates and was found in both Heilongjiang and Fujian. This genotype was first identified in captive wildlife (Malayan sun bear, Tibetian blue bear and Asiatic black bear) from Sichuan, China in 2016 [41]. A variety of wild animals, including Red-Bellied Tree Squirrels, horses, captive Eurasian wild boars, non-human primates, and captive giant pandas were later found to be infected [42–45]. Several studies suggest that bears are a major host for genotype SC02 [12, 13]. System development analysis showed that the genotype has the ability to infect humans, although no reported cases have been documented. Genotype D was found in four bears from Fujian, and this genotype is responsible for most human infections and identified in ≥40 countries [4]. It has been isolated from at least 15 species of animals and some water bodies, and is considered to harbor the highest potential for zoonotic transmission [9, 46]. The occurrence of *E. bieneusi* genotype D in bears suggests its significant zoonotic potential and threat to human health.

## Conclusions

This study demonstrated the occurrence of *Cryptosporidium* and *E. bieneusi* in bears in the Heilongjiang and Fujian Provinces of China and provides molecular characterizations of *C. andersoni* and *Cryptosporidium* rat genotype IV in bears. Genotypes D and SC02 were identified in bears, suggesting their zoonotic potential and threat to human health.

## Methods

### Collection of fecal specimens

From May 2015 to December 2017, a total of 218 fresh fecal specimens (approximately 50 g) were collected from bears in the Heilongjiang (zoo) and Fujian (farm) Provinces of China (Table 1). All fecal specimens were collected from the ground immediately after defecation using a sterile disposable latex glove and each was placed in a labeled sterile bag. To avoid duplicate sampling, each individual animal was identified according to body characteristics such as color and size. The number of collected specimens accounted for approximately 30% of adult or young bears in the farm. All of the specimens were transported to the laboratory in a cooler with ice packs (< 48 h) and stored at 4 °C until processing (< 12 h). The ages of the animals ranged from 1 to 23 years. All animals had no apparent clinical symptoms at the time of sampling.

### DNA extraction

Ten grams of feces of each sample were thoroughly mixed with 30 mL distilled water and filtrates were concentrated by centrifugation at 1500 *g* for 10 min. Genomic DNA was directly extracted from 200 mg of each processed fecal specimen using a QIAamp DNA stool mini kit (QIAgen, Hilden, Germany), according to the manufacturer-recommended procedures (to obtain high yields of DNA, the lysis temperature was increased to 95 °C). Extracted DNA (200 μL) from each sample was transferred to Eppendorf tubes and stored at − 20 °C prior to PCR amplification.

### Genotyping and subtyping of *Cryptosporidium* and *E. bieneusi*

*Cryptosporidium* in the fecal specimens were identified by nested PCR amplification of the SSU rRNA gene fragment of ~ 830 bp and primers and cycle parameters were designed by Xiao et al. (1999) [47]. Meanwhile, all *C. andersoni*-positive isolates were further subtyped through the amplification of four minisatellite/microsatellite markers by nested PCR, respectively, including MS1 coding for hypothetical protein (550 bp), MS2 coding for 90 kDa heat shock protein (450 bp), MS3 coding for hypothetical protein (530 bp), and MS16 coding for leucine rich repeat family protein (590 bp). PCR primers and amplification conditions were performed as previously described [48]. Likewise, all DNA preparations were analyzed for the presence of *E. bieneusi* by amplifying a ~ 390-bp region of the rRNA gene*.* The PCR primers and amplification conditions were performed as previously described [49]. TaKaRa Taq DNA Polymerase (TaKaRa Bio Inc., Tokyo, Japan) was used for all PCR amplifications. Positive chicken-derived *C. bailey* DNA for *Cryptosporidium* and deer-derived BEB6 DNA for *E. bieneusi*) and negative controls (2 μL distilled water) were included in all PCRs. Secondary PCR products were subjected to 1.5% agarose gel electrophoresis and visualized by staining with GelRed (Biotium Inc., Hayward, CA).

### DNA sequencing and analysis

All nested PCR products were sequenced using the same PCR primers used for secondary PCRs on an ABI PRISM™ 3730 DNA Analyzer (Applied Biosystems, Carlsbad, CA, USA), using a BigDye Terminator v3.1 Cycle Sequencing kit (Applied Biosystems). The accuracy of the sequencing data was confirmed by PCR sequencing in both directions. The species or genotypes of *Cryptosporidium* and the genotypes of *E. bieneusi* were identified by comparison to the nucleotide sequences obtained with published GenBank sequences using the Basic Local Alignment Search Tool (BLAST) (http://blast.ncbi. nlm.nih.gov/Blast.cgi) and ClustalX 1.83 (http:// www.clustal.org/), respectively.

### Statistical analysis

Data entry and analysis were performed using Social Sciences (SPSS) 19.0 software. The significance of differences in infection proportions was evaluated using a Pearson’s Chi-square test, whilst the Fisher Exact test was used when more than 20% of cells in the contingency tables had expected frequencies ≤5. *P*-values ≤0.05 were deemed significant. The 95% confidence intervals (95% CI) for prevalence were calculated based on the Poisson distribution.

## Data Availability

All data generated and/or analyzed during this study are included in this published manuscript. The novel *Cryptosporidium* rat genotype IV nucleotide sequence obtained has been deposited in GenBank database under the following accession numbers: MN726617.

## Abbreviations

CI: Confidence intervals
ITS: Internal transcribed spacer
MLST: Multilocus sequence typing
PCR: Polymerase chain reaction
SSU rRNA: Small subunit of nuclear ribosomal RNA
